# Transformation and gene-disruption in the apple-pathogen, *Neonectria ditissima*

**DOI:** 10.1186/s41065-022-00244-x

**Published:** 2022-08-12

**Authors:** Heriberto Vélëz, Jonas Skytte af Sätra, Firuz Odilbekov, Salim Bourras, Larisa Garkava-Gustavsson, Kerstin Dalman

**Affiliations:** 1 Department of Forest Mycology and Plant Pathology, Swedish University of Agricultural Sciences, Box 7026, 75007 Uppsala, Sweden; 2 Department of Plant Breeding, Swedish University of Agricultural Sciences, P.O. Box 190, SE-23422 Lomma, Sweden; 3 Department of Molecular Sciences, Swedish University of Agricultural Sciences, Box 7015, 750 07 Uppsala, Sweden

**Keywords:** European canker, Fruit tree canker, *Neonectria ditissima*, Hydroxyurea, Fungal transformation, GFP

## Abstract

**Background:**

Apple production in Sweden and elsewhere is being threatened by the fungus, *Neonectria ditissima*, which causes a disease known as European canker. The disease can cause extensive damage and the removal of diseased wood and heavily infected trees can be laborious and expensive. Currently, there is no way to eradicate the fungus from infected trees and our knowledge of the infection process is limited. Thus, to target and modify genes efficiently, the genetic transformation technique developed for *N. ditissima* back in 2003 was modified.

**Results:**

The original protocol from 2003 was upgraded to use enzymes currently available in the market for making protoplasts. The protoplasts were viable, able to uptake foreign DNA, and able to regenerate back into a mycelial colony, either as targeted gene-disruption mutants or as ectopic mutants expressing the green fluorescent protein (GFP).

**Conclusions:**

A new genetic transformation protocol has been established and the inclusion of hydroxyurea in the buffer during the protoplast-generation step greatly increased the creation of knockout mutants via homologous recombination. Pathogenicity assays using the GFP-mutants showed that the mutants were able to infect the host and cause disease.

**Supplementary Information:**

The online version contains supplementary material available at 10.1186/s41065-022-00244-x.

## Background

In Sweden, the apple (*Malus x domestica* Borkh.) industry has seen a steady increase in the number of hectares used for apple production, growing by as much as 11% during 2012–2017 [1]. However, apple production in Sweden and elsewhere is being threatened by the fungus *Neonectria ditissima* (Tul. & C. Tul.) Samuels & Rossman, which causes a disease known as European canker [2]. The cool and rainy climate in Sweden favors the fungus, which can cause extensive damage. The diseased wood must be removed and heavily infected trees must be taken out, which leads to a reduction of fruit-bearing area in the orchard combined with an increased workload, resulting in significant economic losses. The removal of entire orchards has been necessary in the USA [3], and in Sweden, where more than 10% of trees can be lost due to canker (Äppelriket, pers. comm.).

*N. ditissima* can infect plants through wounds (e.g., leaf and fruit scars, pruning wounds, broken branches) throughout the year and can cause rotting of fruits during storage [2, 4]. Fungal spores spread aerially and by rain splashing, both within a tree and among nearby trees. Young trees can become infected during propagation [5–7]. Infected branches and the trunk can be girdled, leading to death of all proximal parts [2, 5]. The fungus not only attacks apple and other fruit trees such as pear (*Pyrus communis*) and quince (*Cydonia oblonga*), but also many forest-tree species like *Betula*, *Fagus, and Populus* [3, 8, 9]. *Neonectria*-cankers have been recorded on forest trees in Europe and the USA. In Norway, for example, a *Neonectria* sp. was found to infect subalpine fir (*Abies lasiocarpa*), White fir (*A. concolor*), Siberian fir *(A. sibirica*), and Norway spruce (*Picea abies*) [10]. There are also reports of *Neonectria*-cankers found on forest trees in Sweden, Denmark, United Kingdom, and Belgium [11]. Hence, *Neonectria* species have the propensity to cause damage to European forestry through the extensive scarring and lead to reductions in the quality and value of timber [12].

In Swedish apple orchards, chemical control agents efficient against *N. ditissima* are now prohibited, which underscores the need to breed for resistant apple cultivars. Disease control measures rely primarily on the careful monitoring and removal of infected plants, which is laborious and expensive [2]. Currently, there is no way to eradicate the fungus from infected trees and the available knowledge about the infection process by the fungus is limited.

**Fig. 1:**
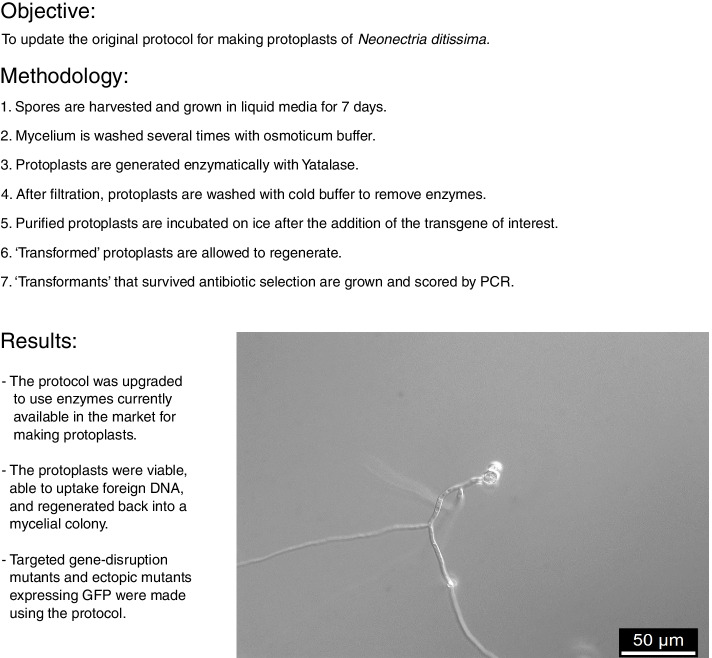
Graphical abstract outlining the protocol steps and results

Recently, the genome of *N. ditissima* has been sequenced and thus far, three draft genomes have been published revealing a genome size of about 44 Mbp [13, 14]. A genetic transformation system using protoplasts of *N. ditissima* was developed in 2003 using a no-longer-available enzyme mixture, called Novozymes 234 [15]. Hence, we set out to develop a new genetic transformation system that, together with the genome information available, will allow for reverse genetic experiments to elucidate the infection process in the future. Here we report on the refinement of the established transformation system, the generation of mutants to express the green fluorescent protein (GFP), and the deletion of AK830_g4721, which encodes a nonribosomal peptide synthetase (NRPS). AK830_g4721 is similar to another NRPS that was shown to be differentially expressed during fungal infection of the fungus *Valsa mali* on apple [16]. Thus, AK830_g4721 was selected for deletion as prove of concept. The steps taken are outlined in Fig. 1. These mutants have the potential to be used on apple-fungal pathogenicity assays to begin elucidating the role of the NRPS and the secondary metabolites produced by the fungus during plant-pathogen interactions.

## Results and discussion

**Fig. 2:**
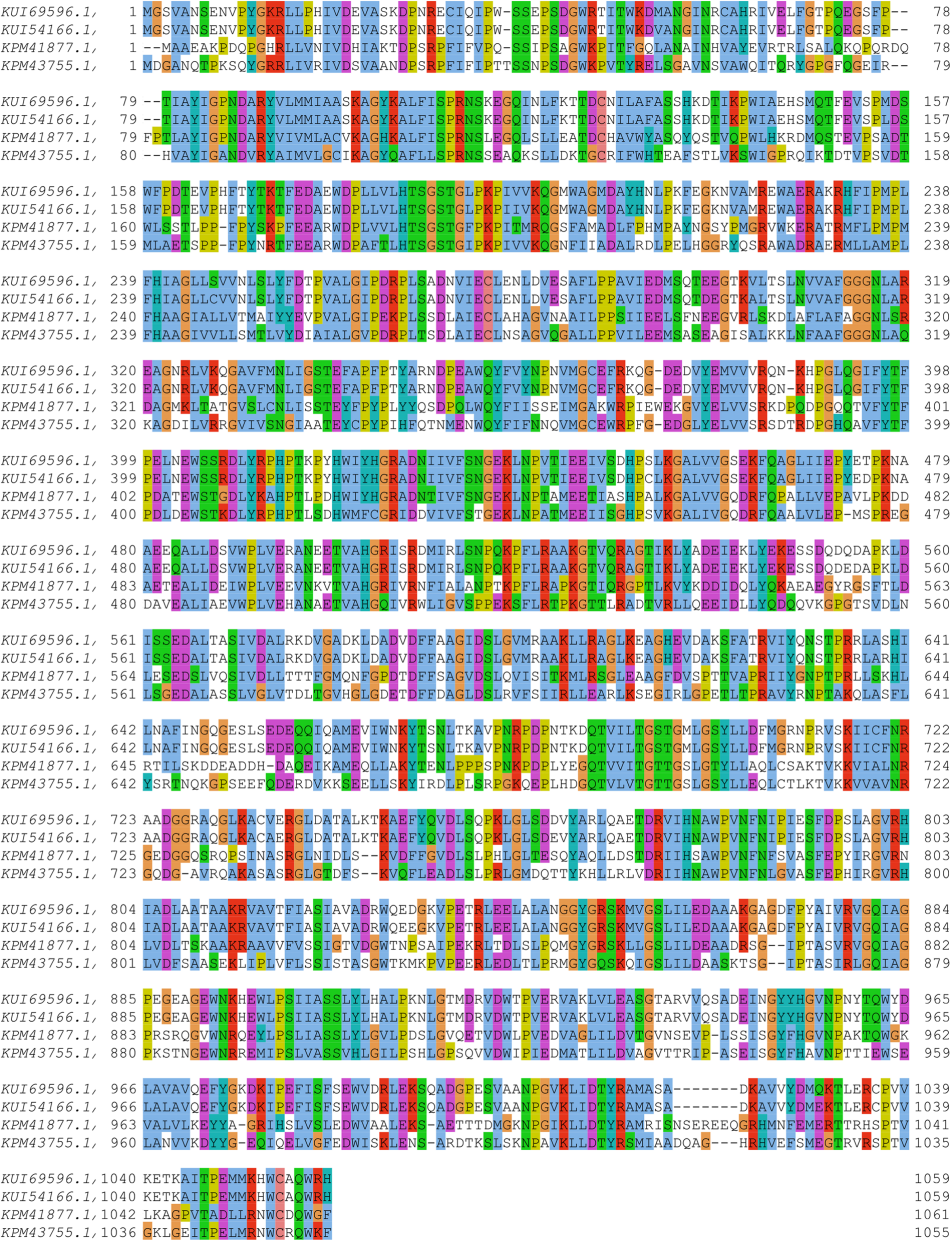
Amino acid sequence alignment of proteins similar to a carboxylic acid reductase from the fungus *Valsa mali* and *V. mali* var. *pyri* (KUI69596.1 and KUI54166.1, respectively), and AK830_g2733 and AK830_g4721 (KPM43755.1) and KPM41877.1, respectively), from *Neonectria ditissima*. Valsa canker on apple, caused by the fungus *V. mali*, is another destructive disease of apples in Eastern Asia. The alignment was generated using Jalview Version 2.11.2.1 [17]. Mutants for AK830_g4721 (KPM41877.1), were generated in this study

Two genes encoding proteins similar to a carboxylic acid reductase from the fungus *V. mali* and *V. mali* var. *pyri* (KUI69596.1 and KUI54166.1, respectively), were found in the genome of *N. ditissima,* AK830_g2733 (KPM43755.1) and AK830_g4721 (KPM41877.1), which were annotated as possible NRPSs*.* The proteins shared 46% amino acid sequence similarity (Fig. 2). To further identify the metabolic products that the NRPS-like enzymes may be responsible for, approximately 50,000 base pairs containing either gene (i.e., AK830_g2733 or AK830_g4721) were analyzed with the online antiSMASH platform [18]. Though the antiSMASH analysis was not able to determine the possible metabolites that are produced by these NRPSs, the presence of genes encoding for transporters, methyltransferases, oxidoreductases, epimerases, transcription factors, etc., would suggest that AK830_g2733 (KPM43755.1) and AK830_g4721 (KPM41877.1) are part of a metabolic cluster (Supplementary Fig. S1).

**Fig. 3:**
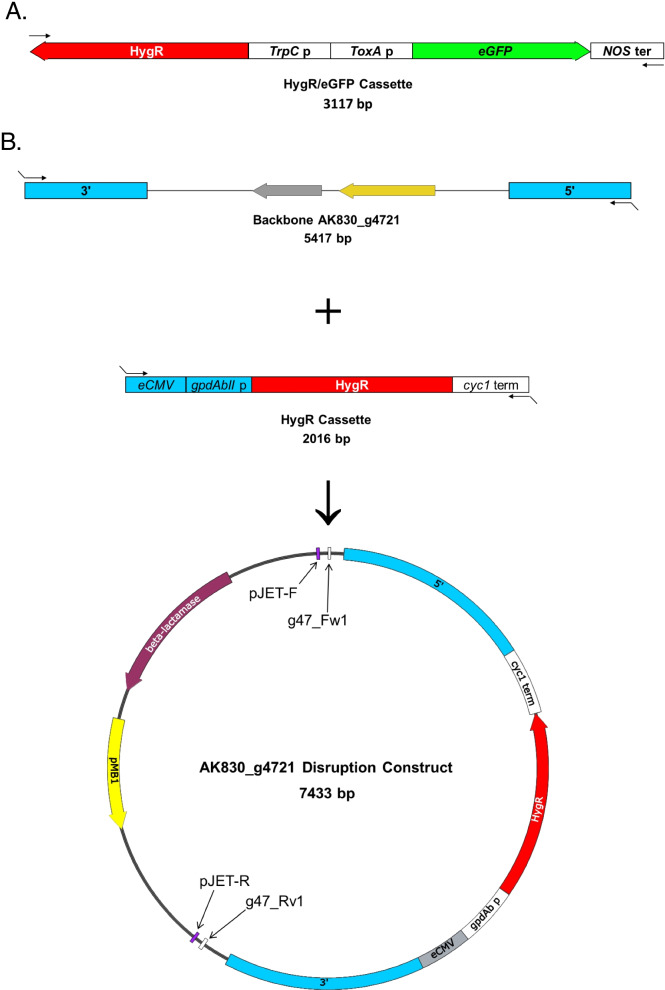
**A** The GFP expression vector, pCT74, developed by Lorang et al., (2001) was used during the transformation. The primers T7 and T3 were used to amplify 3117 bp containing the coding sequence for the GFP expression and hygromycin selection, used during the transformation. **B** Disruption construct generated by GeneArt (Thermo Scientific). Primer Set 1 (Table 2) was used to amplify the coding sequence (3548 bp) from the NRPS gene, AK830_g4721 and cloned into pJet1.2. The backbone of the plasmid with a 5′-fragment (1100 bp) and a 3′-fragment (1200 bp) from the coding sequence at each end, was amplified with Primer Set 3 (Table 2), while Primer Set 4 (Table 2) was used to amplify a linear fragment containing the hygromycin cassette from plasmid pJCA-HygII (Vélëz et al., unpublished). The fragments were ligated using the GeneArt® Seamless Cloning and Assembly Enzyme Mix (Invitrogen). Figure generated using SnapGene® software (Insightful Science; snapgene.com)

**Fig. 4:**
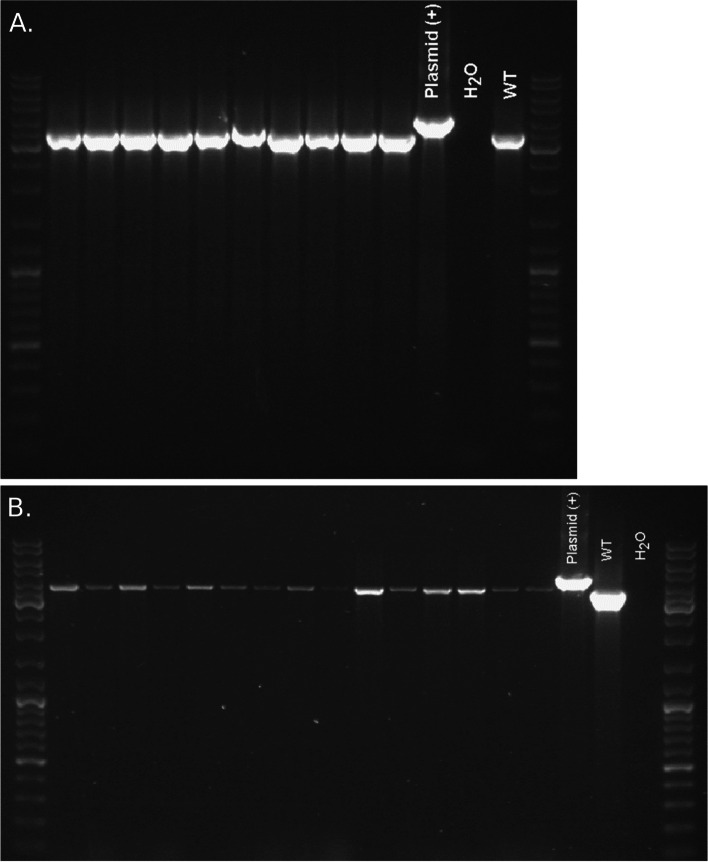
PCR-screen of mutants and wild type *Neonectria ditissima*. Primer Set 1 (Table 2) were used to screen for mutants. A size increase from 3548 bp to 4459 bp would be indicative of a gene disruption. Plasmid DNA, Wild Type genomic DNA, and water were included as controls. A 1-Kb ladder (Thermo Fisher) was included in the first and last lane. **A** Though the mutants grew on the antibiotic hygromycin, all the mutants screened by PCR did not show an increase in the size of the gene. Thus, all the mutants generated were considered ectopic integrants. **B** The mutants grew on the antibiotic hygromycin and showed an increase on the size of the gene, typical of a gene disruption. These mutants were generated by the inclusion of 100 mM hydroxyurea in the protoplast buffer

In this study, we deployed a protoplast-based transformation system based on the initial protocol, which used the enzyme mixture, Novozym 234 [15]. Since this enzyme is no longer available, a new enzyme mixture called Yatalase (Takara) was prepared and tested with and without Lysing Enzymes (L1412 Sigma). Though Sigma has discontinued Lysing Enzymes, there is a new product with a similar name (Lysing Enzymes; NATE-0428) from Creative Enzymes; however, it was not tested in this work. Protoplasts from both tests were viable and able to regenerate. However, protoplasts stored at − 70 °C and used in later transformations were inviable (data not shown). To test the ability of the protoplasts to uptake foreign DNA, PCR products for either ectopic integration (i.e., to express GFP; Fig. 3A) or targeted gene-disruption (i.e., to knockout gene AK830_g4721; Fig. 3B), were used to transform the protoplasts. Selection of transformed and non-transformed protoplast at 25, 30, and 35 μg/ml of hygromycin, showed that 25 μg/ml was enough to kill wild type and select for mutants (data not shown). Nevertheless, individual colonies were always picked from the 35 μg/ml hygromycin-containing plates. Hygromycin-resistant colonies were recovered from three independent experiments, approximately six to 10 days after transformation (Table 1). Approximately, 10 to 50 colonies from gene-knockout experiments were recovered, while there were fewer colonies that regenerated when using only the GFP construct (i.e., ectopic integration); thus, similarly to Tanguay et al. (2003), transformation varied between experiments. However, most of the mutants screened by PCR did not show a gene disruption and therefore, we decided to include 100 mM hydroxyurea in the buffer during the protoplast production step, which has been shown to increase gene targeting in yeasts recalcitrant to transformation [19]. As the authors suggested, the inclusion of hydroxyurea would arrest the protoplast cells in the S/G2 phase, where the homologous recombination pathway predominates [19]. Though the number of protoplasts that regenerated decreased dramatically, all the colonies that were screened after selection showed PCR results consistent with a gene-deletion (Fig. 4). No phenotypic differences were seen between the mutants and the wild type when grown in ASAWA medium (data not shown).

**Table 1:** Summary of mutants generated in this study

	***Gene***	***Colonies picked***	***Gene deletion***
Experiment #1	AK830_g4721T	30	0
Experiment #2	AK830_g4721T	15	0
Experiment #3	AK830_g4721T	20	20
Experiment #1	GFP Transformation	10	
Experiment #2	GFP Transformation	5

**Fig. 5:**
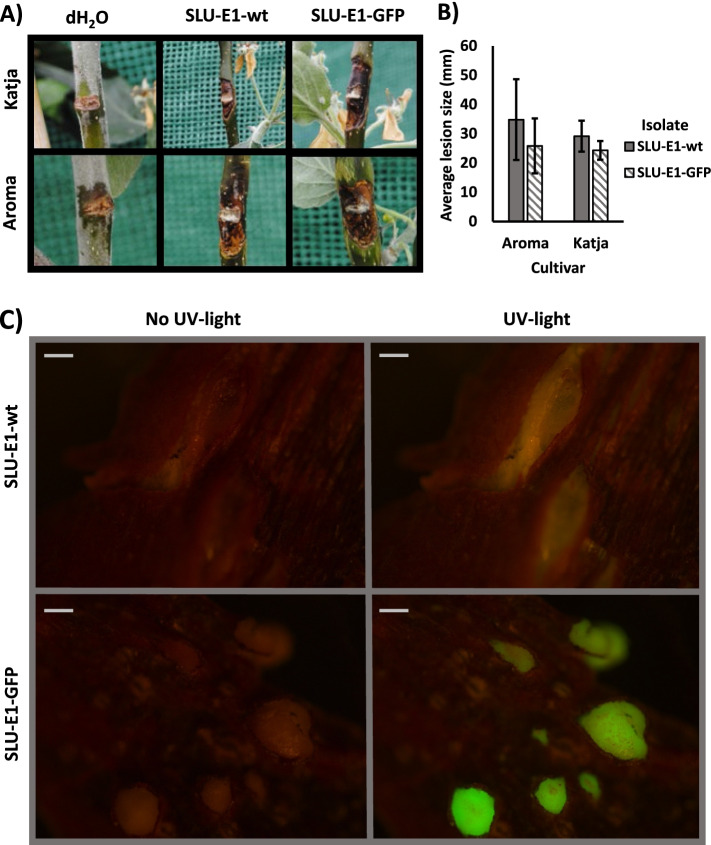
**A** Representative photos of infected wounds 89 DPI on ‘Aroma’ and ‘Katja’ inoculated with distilled water, SLU-E1-wt, and SLU-E1-GFP. **B** Average lesion lengths over 75 and 89 DPI of wounds inoculated with SLU-E1-wt and SLU-E1-GFP, on ‘Aroma’ and ‘Katja’. Error bars designate +/− one standard deviation (*n* = 3 and 6 for wt and GFP, respectively). No pair-wise differences between isolates or cultivars are statistically significant (*p* > 0.05). **C** Infected tissue from the pathogenicity assays visualized under the microscope. The sporodochia from the mutant expressing the green fluorescent protein (SLU-E1-GFP) is clearly seen under UV-light, while the sporodochia from the wild type (SLU-E1-wt) does not have the same intensity (scale bars = 100 μm)

The results of the pathogenicity assays showed that the GFP-transformed isolate SLU-E1-GFP caused symptoms typical for *N. ditissima*, with an identical appearance to the symptoms of the wild type SLU-E1-wt (Fig. 5A). There were no statistically significant differences in average lesion length over 75–89 days post-inoculation (DPI) between the SLU-E1-wt and SLU-E1-GFP inoculated trees (*p* = 0.28 and 0.12 for ‘Aroma’ and ‘Katja’, respectively), and no canker damages were observed on the water-inoculated control trees (Fig. 5A & B). Examination of cankered wood revealed a fluorescent signal in the sporodochia that formed on the SLU-E1-GFP infected necrotic tissue, while only very weak autofluorescence was observed on the SLU-E1-wt infected tissue (Fig. 5C). Similar observations were also true for mycelia and conidia obtained from sporodochia of GFP-transformed and the wt isolate (data not shown).

## Conclusions

We have upgraded the protocol established by Tanguay et al., (2003) to make protoplast of the pathogenic fungus, *N. ditissima*, using currently available enzymes. The protoplasts were viable, able to uptake foreign DNA, and able to regenerate back into a mycelial colony, either as targeted gene-disruption or as ectopic mutants expressing GFP. The inclusion of 100 mM hydroxyurea in the buffer during the protoplast-generation step greatly increased the creation of knockout mutants via homologous recombination. Pathogenicity assays showed that the GFP-mutant’s ability to infect the host and cause disease was unaffected. Thus, these isolates are valuable to future research efforts, providing further insight into the infection process of *N. ditissima*.

## Materials and methods

### Strains and culture conditions

Cultures of *Neonectria ditissima* isolate E1 collected in an orchard close to Lund, Sweden in autumn 2017 from ‘Elise’ (Odilbekov, unpublished), were maintained in Apple Sap Amended Water Agar (ASAWA) media [20]. Liquid Modified Melin Norkrans media (MMN [21]) prepared with sucrose instead of glucose, was used to grow the fungus for genomic DNA extraction or for the growth of mycelial mass for protoplast generation.

#### Identification of the NRPS genes

Blast homology searches using a protein encoding a putative NRPS (KUI69596.1) from the fungus *V. mali* [16], was used to identify the genes encoding a similar NRPSs in the published genome of *N. ditissima* [13].

### DNA extraction, PCR and cloning

DNA was extracted using the NucleoSpin Plant II kit (Macherey-Nagel GmbH & Co. KG, Düren, Germany) and following their protocol. All PCR reactions were done using Phusion Hot Start II DNA Polymerase (Thermo Scientific) following the manufacturers’ protocol, with annealing temperatures at 62 °C. Primers (Table 2) were purchased from Integrated DNA Technologies (Belgium) and dNTPs were purchased from Thermo Scientific. All PCR products were gel-purified using the GeneJET Gel Extraction Kit (Thermo Scientific). pJET 1.2 (Thermo Scientific) and One Shot® TOP10 Chemically Competent *E. coli* (Invitrogen) were used for routine cloning. Plasmids were isolated using the GeneJET Plasmid Miniprep Kit (Thermo Scientific).

**Table 2:** Primers used in this study

	***Name***	***Sequence(5’→3’)***	***Description***
***Set 1***	g47_Fw1	atccagtaggccatcgttcc	Amplify NRPS AK830_g4721 and
	g47_Rv1	ctggtgatgtgtccgtggat	Screen for knockouts
***Set 2***	g47_Fw2	ctttaccttacaatggcggcag	Amplify NRPS AK830_g4721
	g47_Rv2	gatcacaccagttcctcagca	
***Set 3***	Backboneg47_Fw	ccattgacgactgtggtcattactgg	Amplify NRPS AK830_g4721 cloned in pJet1.2
	Backboneg47_Rv	gaatcatgagtaaggggtactaactc	
***Set 4***	Insertg47_Hyg_Fw	cccttactcatgattcgcacaaaaaaa	Amplify Hygromycin cassette
	Insertg47_Hyg_Rv	ccacagtcgtcaatgggtggagt	
***Set 5***	T3	gcaattaaccctcactaaagg	Amplify GFP/Hygromycin cassette from pCT74
	T7	taatacgactcactataggg	

### Knock-out construct generation

Primer Set 1 (Table 2) was used to amplify the coding sequence (3548 bp) from the NRPS gene AK830_g4721. After gel purification, the PCR product was cloned into pJet1.2 and transformed into One Shot® TOP10 Chemically Competent *Escherichia coli* (Invitrogen). Positive clones were verified by restriction digest. A positive clone from the plasmid prep was used as a template in a PCR reaction with Primer Set 3 (Table 2) to amplify a linear fragment comprised of the backbone of the plasmid flanked with a 5′-fragment (1100 bp) and a 3′-fragment (1200 bp) from the coding sequence at each end (Fig. 3). Primer Set 4 (Table 2) was used to amplify the hygromycin cassette from plasmid pJCA-HygII (Vélëz et al., unpublished). After gel purification, both PCR fragments were used with GeneArt® Seamless Cloning and Assembly Enzyme Mix (Invitrogen) by following the manufacturers’ protocol and at the end of the 30 min incubation step, 5 μl were transformed into One Shot® TOP10 Chemically Competent *E. coli* (Invitrogen). Positive clones were verified by restriction digest.

### PCR products for transformation

A PCR fragment containing the assembled construct (Fig. 3B), was amplified from a positive clone using primer Set 2, purified using the E.Z.N.A.® Cycle-Pure Kit (Omega Bio-tek), and used for the transformation of protoplasts to make AK830_g4721-knockouts. Similarly, primer Set 5 (Table 2) was used to amplify the coding sequence (3117 bp) for the GFP/Hygromycin cassette from plasmid pCT74 [22] (Fig. 3A), purified using the E.Z.N.A.® Cycle-Pure Kit (Omega Bio-tek), and used for the transformation of protoplasts to make GFP-expressing mutants.

### Protoplast production and transformation

To produce protoplasts, the protocol by Tanguay et al. (2003) was followed with several modifications. Spores (10^9^) were transferred to a 250 ml Erlenmeyer flask containing 100 ml of liquid MMN media and incubated at 25 °C for 4 days without agitation. The fungal mycelium was transferred to a 50 ml conical tube and pelleted by centrifugation at 3000 x g at 4 °C for 10 min. After decanting the growth media, the mycelium was rinsed with osmoticum buffer (OB; 0.8 M NaCl; 50 mM Maleate Buffer pH 5.5) and centrifuged as before, repeating this step once again. Finally, the pellet was re-suspended in 4 ml of filtered-sterilized (0.22 μm filter, Sarstedt) lysing buffer (75 mg/ml BSA; 10 mg/ml Yatalase (Takara); 5 mg/ml Lysing Enzymes (L1412 Sigma); 5 mg/ml L-cysteine dissolved in OB), vortexed twice for 30 sec to mix, and incubated for 1.5 hr. in a water bath at 30 °C with gentle shaking. When the protoplasts were intended for the generation of gene disruption, 100 mM hydroxyurea was included in the lysing buffer prior to the filter-sterilizing step. If the protoplasts were intended for the generation of ectopic integration, the hydroxyurea was omitted. The protoplasts were separated from cell wall debris by filtration through four layers of sterile Precision Wipes (Kimtech Science, Kimberly-Clark) into a new 50 ml conical tube. The protoplasts were washed twice, with 20 ml of ice cold STC buffer [1 M d-sorbitol, 50 mM Tris–HCl (pH 8.0), 200 mM CaCl_2_], and pelleted by centrifugation at 2500 x g at 4 °C for 8 min each time. The protoplasts were re-suspended in STC and their concentration adjusted accordingly. When the protoplasts were intended for the generation of gene disruption, 40 mM hydroxyurea was also included in the STC buffer. For the transformation, 100 μl of protoplasts (10^8^ in STC) aliquoted in 50 ml conical tubes (Sarstedt), were incubated on ice for 30 min after the addition of 10 μg of PCR’d-DNA construct in 40 μl of STC and 10 μl of Spermidine (10 mM dissolved in STC and filtered-sterilized). After 30 minutes, 600 μl of PTC buffer [40% (w/v) PEG 4000, 50 mM Tris-HCl (pH 8.0), 200 mM CaCl_2_] was added dropwise to the tubes and incubated at room temperature for an additional 30 min. The volume was brought up to 15 ml with STC (without hydroxyurea) and centrifuged as before to remove the PEG. After the liquid was removed by aspiration, liquid MYGS (1% maltose, 0.04% glucose, 0.04% yeast extract, and 0.7 M sucrose) was added to the 20 ml mark and placed at 28 °C for 24 hrs. The following day, each 50 ml conical tube was subdivided into two 10 ml-aliquots and each aliquot was mixed with 30 ml of ASAWA media (containing 0.7 M sucrose and 1% (w/v) low melting point agarose), 35 μg/ml of Hygromycin B (InvivoGen, France) and poured into big plates (150 ϕ; Sarstedt) after gentle mixing. Plates were incubated at 28 °C for 10 days in the dark and putative transformants (selected as single colonies) were transferred to ASAWA plates. Visualization of GFP-expressing mutants was done using a Leica DM5500B microscope equipped with a DFC360fx camera.

### Pathogenicity assays

Pathogenicity assays were conducted on potted trees of two cultivars differing in their levels of resistance: the highly susceptible cultivar ‘Katja’, and ‘Aroma’ known as having a high level of resistance [23, 24]. The trees were produced in a Swedish nursery in March 2018 and inoculated in an unheated greenhouse taking all the necessary precautions. The trees (six and three trees per cultivar, for the GFP- and wt-isolate, respectively) were inoculated in manually-inflicted wounds as previously described [24], except that five wounds per tree were inoculated instead of three. Inoculum of wild type and GFP-transformed *N. ditissima* isolate SLU-E1 was produced on ASAWA media [20], and each wound was inoculated with 1000 conidia in 10 μl water. Prior to the inoculation, spores of the GFP-transformed isolate were checked for the green fluorescent signal. One tree of each cultivar was inoculated with distilled water as a control.

After inoculations, the trees were kept at 16 ± 2 °C and 70% relative humidity. At 75 and 89 DPI, the trees were examined for the symptoms typical of *N. ditissima*. The lesions were measured with a digital caliper. Both GFP- and wt-inoculated symptomatic-wood was examined in a Leica DMLB light microscope equipped with a Leica DFC450 C camera and a Prior Lumen200 UV-light source. In addition, water-dispersed mycelium and conidia were collected from the symptomatic wood and examined.

### Statistical analyses

Statistical analysis was performed in base R [25]. The average lesion length over the five technical replicates per tree was used for pairwise comparisons between cultivars for each isolate, and between isolates for each cultivar, respectively. Trees were treated as replicates with either a Student’s t-test or a Welch’s t-test used to test for significant differences, depending on whether the two groups had significantly different variances or not based on an F test. Both wt and GFP inoculated samples of ‘Aroma’ had 7–9 fold larger variances than the corresponding ‘Katja’ sample. Lesions shorter than five mm 75 DPI were treated as missing values. Bar plots were generated in Microsoft Excel. As none of the trees inoculated with water developed symptoms, these were excluded from the analysis.

## Supplementary Information

**Additional file 1 **: **Supplementary Figure S1.** A). Domain similarity between the nonribosomal peptide-synthetases encoded by *Valsa mali* (KUI69596.1) and *Neonectria ditissima* (KPM43755.1 and KPM41877.1). B). The antiSMASH analysis showed the presence of genes in clusters (e.g., methyltransferases, oxidoreductases, epimerases, transcription factors, etc.), that are associated with the production of secondary metabolites. Figure generated using SnapGene® software (Insightful Science; snapgene.com).

## Data Availability

The datasets supporting the conclusions of this article are included within the article. Materials are available from the corresponding author on reasonable request.

## Abbreviations

GFP: Green fluorescent protein
NRPS: Nonribosomal peptide synthetase
S (phase): Synthesis Phase
G2 (phase): Gap 2 phase or Growth 2 phase
DPI: Days post-inoculation
ASAWA: Apple Sap Amended Water Agar
MMN: Modified Melin Norkrans media
dNTPs: Deoxyribonucleotide triphosphates
bp: Base pairs
min: Minutes
OB: Osmoticum buffer
STC: Sorbitol Tris-HCl CaCl_2_
wt: Wild type

## References

[1] Fruktträd PJ. Statistiska meddelanden JO 33 SM 1802. Sveriges officiella statistik, Statens Jordbruksverk; 2017.https://jordbruksverket.se/om-jordbruksverket/jordbruksverkets-officiella-statistik/jordbruksverkets-statistikrapporter/statistik/2020-06-18-frukttrad-2017#h-Rapport.

[2] Weber RWS (2014). Biology and control of the apple canker fungus *Neonectria ditissima* (syn. *N. Galligena*) from a northwestern European perspective. Erwerbs-Obstbau..

[3] Jones AL, Aldwinckle HS (1990). Compendium of apple and pear diseases.

[4] Xu XM, Robinson JD (2010). Effects of fruit maturity and wetness on the infection of apple fruit by *Neonectria galligena*. Plant Pathol.

[5] Ghasemkhani M, Sehic J, Ahmadi-Afzadi M, Nybom H, Garkava-Gustavsson L (2015). Screening for partial resistance to fruit tree canker in apple cultivars. Acta Hortic.

[6] Børve J, Kolltveit SA, Talgø V, Stensvand A (2018). Apple rootstocks may become infected by *Neonectria ditissima* during propagation. *Acta Agriculturae Scandinavica, section B — Soil & Plant*. Science..

[7] Wenneker M, de Jong PF, Joosten NN, Goedhart PW, Thomma BPHJ (2017). Development of a method for detection of latent European fruit tree canker (*Neonectria ditissima*) infections in apple and pear nurseries. Eur J Plant Pathol.

[8] Gómez-Cortecero A, Saville RJ, Scheper RWA, Bowen JK, Agripino De Medeiros H, Kingsnorth J (2016). Variation in host and pathogen in the Neonectria/Malus interaction; toward an understanding of the genetic basis of resistance to European canker. Front Plant Sci.

[9] Walter M, Glaister MK, Clarke NR, Von Lutz H, Eld Z, Amponsah NT (1975). Are shelter belts potential inoculum sources for *Neonectria ditissima* apple tree infections?. New Zealand Plant Protection..

[10] Talgø V (2012). Neonectria-canker on trees in Norway. J Agricult Ext Rural Dev.

[11] Schmitz S, Charlier A, Chandelier A (2017). First report of *Neonectria* neomacrospora on *Abies grandis* in Belgium. New Disease Reports.

[12] Metzler B, Meierjohann E, Kublin E, Von Wühlisch G (2002). Spatial dispersal of *Nectria ditissima* canker of beech in an international provenance trial. For Pathol.

[13] Gómez-Cortecero A, Harrison RJ, Armitage AD (2015). Draft genome sequence of a European isolate of the apple canker pathogen, Neonectria Ditissima. Genome Announc.

[14] Deng CH, Scheper RWA, Thrimawithana AH, Bowen JK (2015). Draft genome sequences of two isolates of the plant-pathogenic fungus *Neonectria ditissima* that differ in virulence. Genome Announc.

[15] Tanguay P, Coupal J, Bernier L (2003). Genetic transformation, electrophoretic karyotyping and isolation of insertional mutants in the tree pathogenic fungus *Neonectria galligena*. For Pathol.

[16] Ke X, Yin Z, Song N, Dai Q, Voegele RT, Liu Y (2014). Transcriptome profiling to identify genes involved in pathogenicity of *Valsa Mali* on apple tree. Fungal Genet Biol.

[17] Waterhouse AM, Procter JB, Martin DMA, Clamp M, Barton GJ (2009). Jalview version 2-a multiple sequence alignment editor and analysis workbench. Bioinformatics..

[18] Blin K, Medema MH, Kazempour D, Fischbach MA, Breitling R, Takano E (2013). AntiSMASH 2.0--A versatile platform for genome mining of secondary metabolite producers. Nucleic Acids Res.

[19] Tsakraklides V, Brevnova E, Stephanopoulos G, Shaw AJ (2015). Improved gene targeting through cell cycle synchronization. PLoS One.

[20] Amponsah NT, Walter M, Scheper RWA (2014). Agar media for isolation of *Neonectria ditissima* from symptomatic and asymptomatic apple tissues and production of infective conidia. New Zealand Plant Protection.

[21] Baar J, Comini B, Elferink MO, Kuyper TW (1997). Performance of four ectomycorrhizal fungi on organic and inorganic nitrogen sources. Mycol Res.

[22] Lorang JM, Tuori RP, Martinez JP, Sawyer TL, Redman RS, Rollins J (2001). Green fluorescent protein is lighting up fungal biology. Appl Environ Microbiol.

[23] Garkava-Gustavsson L, Zborowska A, Sehic J, Rur M, Nybom H, Englund J-E (2013). Screening of apple cultivars for resistance to European canker, Neonectria ditissima. Acta Horticulturae.

[24] Garkava-Gustavsson L, Ghasemkhani M, Zborowska A, Englund JE, Lateur M, Van De Weg E (2016). Approaches for evaluation of resistance to European canker (*Neonectria ditissima*) in apple. Acta Hortic.

[25] R Core Team. R (2013). A language and environment for statistical computing.

