# Supplementary figures and images for: Transformation and gene-disruption in the apple-pathogen, Neonectria ditissima
Source: Hereditas. 2022 Aug 12;159:31. doi: 10.1186/s41065-022-00244-x (PMC9373326; doi:10.1186/s41065-022-00244-x)

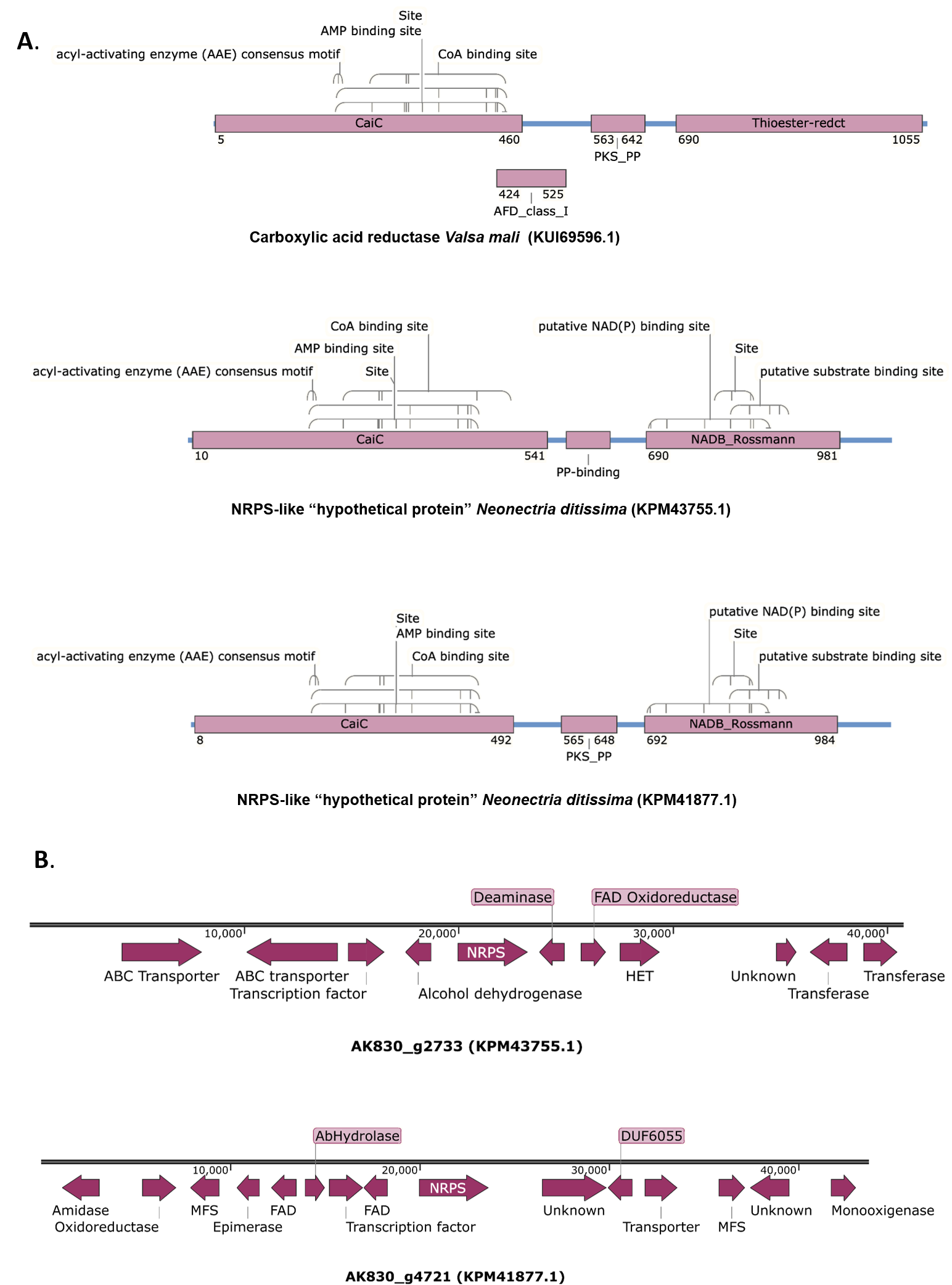

Supplement: Supplementary file 1 — Additional file 1 : Supplementary Figure S1. A). Domain similarity between the nonribosomal peptide-synthetases encoded by Valsa mali (KUI69596.1) and Neonectria ditissima (KPM43755.1 and KPM41877.1). B). The antiSMASH analysis showed the presence of genes in clusters (e.g., methyltransferases, oxidoreductases, epimerases, transcription factors, etc.), that are associated with the production of secondary metabolites. Figure generated using SnapGene® software (Insightful Science; snapgene.com). [file 41065_2022_244_MOESM1_ESM.tif]
